# Screening of Atrial Fibrillation in Cryptogenic Ischemic Stroke—Does Timing Make the Difference?

**DOI:** 10.1111/ene.70526

**Published:** 2026-03-25

**Authors:** Olli P. Suomalainen, Jukka Putaala

**Affiliations:** ^1^ Department of Neurology, Helsinki University Hospital and Clinical Neurosciences University of Helsinki Helsinki Finland

Cardioembolic causes account for roughly a quarter of all ischemic strokes (IS) [1]. Of them, known atrial fibrillation (AF) is the most frequently documented source for embolism, and its share of stroke etiologies is likely to be increased along an aging population as the incidence of AF strongly increases with age [1]. Anticoagulation with direct oral anticoagulants in patients with AF is both effective and safe, making AF detection clinically highly relevant in patients with IS [1]. In approximately one‐sixth of IS patients, the etiology of stroke remains cryptogenic [2]. The European Stroke Organization recommends a minimum of 48–72 h of AF screening in patients with cryptogenic ischemic stroke (CIS) and the recommendation is even longer according to the American Heart Association (AHA) guidelines (up to several weeks) [3, 4]. In patients with embolic stroke of undetermined source (ESUS), a subtype of CIS, occult AF has been thought to be the most important cryptogenic source of embolism [2]. However, the large randomized clinical trials (RCTs) like NAVIGATE‐ESUS and RESPECT‐ESUS have failed to demonstrate the efficacy and safety of anticoagulation even in high‐risk patients without detection of AF [1, 5].

For the time being, there is no clear consensus how intensively, and by which screening methods the patients with CIS or especially with ESUS should be screened for AF [6]. The potential screening methods range from handheld and wearable devices (up to few weeks of intermittent, event‐triggered or continuous monitoring) to implantable cardiac monitors (ICM), which can monitor patients up to few years and potentially measure AF burden [7]. ICMs have shown a variable detection rate of up to 70% in patients with CIS [8, 9, 10]. Some national guidelines recommend more intensive AF screening in high‐risk patients with potential AF‐related risk factors like atrial tachycardia, frequent atrial extrasystoles, and high natriuretic pro‐peptide like proBNP [11]. Especially, the latter has showed efficacy as a high‐risk biomarker in AF screening [12]. As the causality of detected AF paroxysm after IS might be unclear, especially at later screening stages, recent studies have suggested that ultrafast screening approach after CIS could be more relevant and increase causality of AF detected soon after stroke (AFDAS) more accurately than later screening [10]. As there often are limited resources regarding AF screening even with ESUS patients, the timing and focus of AF screening in high‐risk patients would seem clinically highly relevant.

Given this context, we read with great interest the article entitled “Ultra‐Early Continuous ECG Monitoring for Silent Atrial Fibrillation Diagnosis in Cryptogenic Stroke: The CRIPTOFAST Randomized Controlled Trial” by Vallès E. et al. in this issue of *European Journal of Neurology* (Volume 33, Issue 1, https://doi.org/10.1111/ene.70482). CRIPTOFAST RCT is a randomized, controlled, parallel‐arm, open‐label single‐center study which shows that ultrafast AF screening in CIS patients can be efficient and feasible. CRIPTOFAST included 59 patients, of which 52.5% patients were randomized into ultrafast ICM implantation and 47.5% patients to standard care after > 48 h of baseline screening. Paroxysmal AF was detected in 25% of patients, with a significantly higher incidence in the ICM group (43.3%) compared to the control group (7.1%). Most of the AF episodes were detected within the first 100 days since ICM implantation with the maximum peak within the first 30 days. The median AF burden was 9% of the total monitoring time in the ICM group, and the median duration of the first AF episode was 17 min (ranging from 4 min to almost 24 h). This median duration is also longer than 5 min which is a threshold to a clinically significant AF paroxysm according to the AHA guidelines. Low glomerular filtration rate was the only clinical biomarker significantly associated with the diagnosis of paroxysmal AF. The presence of subtle atrial abnormalities was strongly associated with a higher incidence of silent AF although neither the left ventricular ejection fraction nor any of the left atrial measurements like left atrial dilatation, which have been shown to be associated with AF in CIS patients [1, 12], were nonsignificant, likely due to the small sample size despite a positive trend.

The study by Vallès E et al. is an important contribution, as there are no RCTs regarding ultrafast approaches in CIS patients to date [8, 10]. The study also assessed AF burden, which is a highly relevant biomarker, as the causality of the detected AF paroxysms with CIS may be potentially more accurately evaluated. Despite the highly relevant results, the CRIPTOFAST study does have limitations affecting translation to clinical practice. The sample size is small, leading to nonsignificant results in AF risk biomarkers, especially regarding proBNP. Despite most of the included patients (83%) having ESUS‐type IS in which the AF screening is previously considered to be clinically relevant, the study also included patients with transient ischemic attack [2]. The results of the CRIPTOFAST study still imply that the AF screening in CIS patients should be performed in a timely manner, potentially with ICMs right after the index event in high‐risk patients.

The results of the CRIPTOFAST study align with a recent meta‐analysis by D'Anna et al. [8], showing that early implantation of ICM (< 31.5 days from index event) resulted in a significantly higher yield of new AF compared to later implantation (30.0% vs. 23.7%; *p* = 0.0017), independent of monitoring duration. These results suggest that AF detection in CIS should be done vigorously early after the index stroke in CIS patients. However, given the high cost of screening every CIS patient with ICMs, fast, preferably step‐by‐step baseline screening approaches after the index CIS should be assessed systematically. Such approaches could utilize alternative first‐line methods like handheld and wearable devices, as observational data suggests a reasonable yield for them and their use is even suggested by some national guidelines [9, 11]. Therefore, larger, preferably multicenter RCTs are needed to compare different ultrafast AF screening strategies. Finally, AFDAS might not be persistent after IS and there is also a need for research regarding the causality of poststroke detected AF.

## Author Contributions

**Olli P. Suomalainen:** writing – review and editing. **Jukka Putaala:** writing – review and editing.

## Conflicts of Interest

The authors declare no conflicts of interest.

## Linked Article

Ultra‐Early Continuous ECG Monitoring for Silent Atrial Fibrillation Diagnosis in Cryptogenic Stroke: The CRIPTOFAST Randomized Controlled Trial. https://doi.org/10.1111/ene.70482.

## Data Availability

Data sharing not applicable to this article as no datasets were generated or analysed during the current study.
